# Closed Genome Sequence of Bacillus paralicheniformis Strain CBMAI 1303, a Bacterium Applied for Phytopathogen Biocontrol

**DOI:** 10.1128/MRA.01507-18

**Published:** 2019-01-17

**Authors:** Victor Satler Pylro, Armando Cavalcante Franco Dias, Fernando Dini Andreote, Alessandro de Mello Varani, Laura Rabelo Leite, Cristiane Cipolla Fasanella Andreote, Eduardo Roberto de Almeida Bernardo, Thales Facanali Martins

**Affiliations:** a Department of Biology, Federal University of Lavras (UFLA), Lavras, Minas Gerais, Brazil; b Andrios Assessoria, Piracicaba, São Paulo, Brazil; c Soil Microbiology Laboratory, Luiz de Queiroz College of Agriculture, University of São Paulo (ESALQ/USP), Piracicaba, São Paulo, Brazil; d Departamento de Tecnologia, Faculdade de Ciências Agrárias e Veterinárias, Universidade Estadual Paulista (UNESP), Jaboticabal, São Paulo, Brazil; e Progenética, Hermes Pardini, Vespasiano, Minas Gerais, Brazil; f Agrivalle Brasil Indústria e Comércio de Produtos Agrícolas Ltda, Salto, São Paulo, Brazil; Queens College

## Abstract

We report the complete genome sequence and annotation of Bacillus paralicheniformis strain CBMAI 1303, a bacterium applied for phytopathogen biocontrol.

## ANNOUNCEMENT

Biological control with bacteria shows great potential as an eco-friendly and feasible alternative to replace, or at least diminish, the application of chemicals for pathogen control (1). Understanding the mechanisms involved in the interaction between any biological control agent and a target phytopathogen is crucial for enhancing and extending the use of these organisms in agriculture. To gain insight into the use of Bacillus paralicheniformis strain CBMAI 1303, a Gram-positive and rod-shaped soil bacterium used for biological control of phytopathogens, we sequenced the genome using the MiSeq platform (Illumina, USA). Several closed related *Bacillus* spp. have been used for phytopathogen biocontrol, including Magnaporthe grisea in rice (2) and Botrytis cinerea in tomato (3). Moreover, a preliminary study by our group found that B. paralicheniformis strain CBMAI 1303 has the potential to control *Rhizoctonia* sp. and *Colletotrichum* sp. growth. This isolate was obtained from the Coleção Brasileira de Micro-organismos de Ambiente e Indústria (CBMAI; http://cbmai.cpqba.unicamp.br) and cultivated in nutrient agar, and the genomic DNA was extracted using the Wizard genomic DNA purification kit (Promega), following the manufacturer’s instructions.

Paired-end sequencing libraries (2 × 250 bp) were constructed with the Nextera XT kit (Illumina), following the manufacturer’s instructions. After quality filtering with Trimmomatic version 0.33 (4) (parameters PE; TRAILING:10 LEADING:10 SLIDINGWINDOW:4:10), we obtained 1,537,492 reads consisting of approximately 80× genome coverage. All reads were *de novo* assembled with the SPAdes genome assembler version 3.12 (5), following default parameters for Illumina data, which generated 847 contigs. Contigs with a length less than 200 bp were discarded, which resulted in a genome sequence assembled into 67 contigs. Scaffolds were generated with the reference-based scaffolder MeDuSa (6) with Bacillus paralicheniformis ATCC 9945a (GenBank accession number NC_021362) as a guide for alignment, which resulted in four scaffolds. A proposed genome consensus was manually closed by comparisons against the closest reference genome (Bacillus paralicheniformis ATCC 9945a), paired-end read mapping, and a BLAST analysis. Genome completeness and contamination were estimated using CheckM (7) in the lineage-specific mode. Genome statistics were estimated with QUAST (8), using Bacillus paralicheniformis ATCC 9945a as the reference genome. The average nucleotide identity based on BLAST (ANIb) between our genome and its reference was determined by JSpecies (7); ANI scores greater than 95% indicate that they belong to the same species (9, 10).

The final genome sequence consists of a single contiguous circular chromosome. The estimated genome size is 4,476,034 bp, with a G+C content of 45.9%. The genome completeness estimated by CheckM was 99.41%, and the contamination was 0.05%, which classifies it as nearly complete and with low contamination. The genome annotation was performed with PATRIC version 3.5.23 (11), which identified 4,752 coding sequences (CDS), 93 predicted noncoding RNAs (72 tRNAs and 21 rRNAs, encompassing 7 rRNA operons), and 42 repeated regions. The calculated ANIb was 99.89% between Bacillus paralicheniformis CBMAI 1303 and Bacillus paralicheniformis ATCC 9945a, which classified them as belonging to the same species.

### Data availability.

This whole-genome shotgun project has been deposited at DDBJ/EMBL/GenBank under the accession number CP033389. The version described in this paper is the first version, CP033389.1. Raw reads are available under the BioProject accession number PRJNA509356.
